# Association between NRGN gene polymorphism and resting-state hippocampal functional connectivity in schizophrenia

**DOI:** 10.1186/s12888-019-2088-5

**Published:** 2019-04-05

**Authors:** Yifan Zhang, Xiaohong Gong, Zhiyang Yin, Lingling Cui, Jian Yang, Pengshuo Wang, Yifang Zhou, Xiaowei Jiang, Shengnan Wei, Fei Wang, Yanqing Tang

**Affiliations:** 1 Department of Psychiatry, The First Affiliated Hospital of China Medical University, Shenyang, Liaoning 110001 People’s Republic of China; 2 State Key Laboratory of Genetic Engineering and MOE Key Laboratory of Contemporary Anthropology, School of Life Sciences, Fudan University, Shanghai, 200433 People’s Republic of China; 3 Department of Radiology, The First Affiliated Hospital of China Medical University, Shenyang, Liaoning 110001 People’s Republic of China; 4 Department of Psychiatry and Gerontology, The First Affiliated Hospital of China Medical University, 155 Nanjing North Street, He ping District, Shenyang, Liaoning 110001 People’s Republic of China; 5 Brain Function Research Section and Department of Psychiatry and Radiology, The First Affiliated Hospital of China Medical University, 155 Nanjing North Street, He ping District, Shenyang, Liaoning 110001 People’s Republic of China

**Keywords:** Schizophrenia, NRGN, Hippocampus, fMRI, Functional connectivity

## Abstract

**Background:**

Based on genome-wide association studies, a single-nucleotide polymorphism in the NRGN gene (rs12807809) is considered associated with schizophrenia (SZ). Moreover, hippocampal dysfunction is associated with rs12807809. In addition, converging evidence suggests that hippocampal dysfunction is involved in SZ pathophysiology. However, the association among rs12807809, hippocampal dysfunction and SZ pathophysiology is unknown. Therefore, this study investigated the association between rs12807809 and hippocampal functional connectivity at rest in SZ.

**Methods:**

In total, 158 participants were studied, including a C-carrier group carrying the non-risk C allele (29 SZ patients and 46 healthy controls) and a TT homozygous group carrying the risk T allele (30 SZ patients and 53 healthy controls). All participants were scanned using resting-state functional magnetic resonance imaging. Hippocampal functional connectivity was computed and compared among the 4 groups.

**Results:**

Significant main effects of diagnosis were observed in the functional connectivity between the hippocampus and bilateral fusiform gyrus, bilateral lingual gyrus, left inferior temporal gyrus, left caudate nucleus, bilateral thalamus and bilateral anterior cingulate gyri. In contrast, no significant main effect of genotype was found. In addition, a significant genotype by diagnosis interaction in the functional connectivity between the hippocampus and left anterior cingulate gyrus, as well as bilateral middle cingulate gyri, was observed, with TT homozygotes with SZ showing less functional connectivity than C-carriers with SZ and healthy control TT homozygotes.

**Conclusions:**

These findings are the first to suggest an association between rs12807809 and abnormal Papez circuit function in patients with SZ. This study also implicates NRGN variation and abnormal Papez circuit function in SZ pathophysiology.

**Electronic supplementary material:**

The online version of this article (10.1186/s12888-019-2088-5) contains supplementary material, which is available to authorized users.

## Background

Schizophrenia (SZ) is a severe mental disorder with a lifetime prevalence of approximately 1%. Genetic factors are an important part of the aetiology and pathogenesis of SZ [1]. In recent years, in genome-wide association studies, a single nucleotide polymorphism (SNP), rs12807809, in the NRGN gene was reportedly associated with SZ [2], and the T allele conferred a high risk for SZ. An association of rs12807809 with SZ was also found in 1005 SZ patients and 1069 controls in a South Indian population; further analysis found a moderate association of rs12807809 with flat affect and hallucinations [3]. In addition, an expression quantitative trait loci (eQTL) analysis provided evidence for an increased risk for SZ with rs12807809 at a molecular level [4]. Over the years, rs12807809 has been extensively studied; these studies have suggested that rs12807809 is associated with structural and functional abnormalities in the brain and symptom severity in SZ patients [5–10].

The NRGN gene is the human homologue of the rat RC3/Ng gene, which is localised to chromosome 11q24.2, spans approximately 12 kb and contains 4 exons and 3 introns [11]. A 78-amino-acid protein is encoded by exon 1 and exon 2. Exon 3 and exon 4 comprise untranslated sequences. Moreover, intron 1 contains a thyroid hormone-responsive element [12]. NRGN encodes a postsynaptic protein kinase C (PKC) substrate, which is expressed in the soma and dendrites of neurons in the hippocampus and striatum and in the cerebral cortex [13–16]. This protein is called neurogranin (Ng), given that its immunoreactivity is correlated with granule-like structures in hippocampal pyramidal cells in electron micrographs [17]. The phosphorylation of Ng by PKC is activated by Ca^2+^, phospholipids, and diacylglycerol. In the absence of Ca^2+^, Ng binds to calmodulin (CaM), which also regulates neuromodulin. Moreover, the phosphorylation of Ng by PKC is inhibited by CaM [18]. In the presence of Ca^2+^, the affinity of Ng binding to CaM is decreased. Ng is considered to regulate many postsynaptic signal transduction pathways due to the role of Ng in regulating Ca^2+^ and CaM. Ng knockout mice showed performance deficits in a spatial learning paradigm. In addition, Ng knockout mice exhibited changes in synaptic plasticity, which suggests the involvement of Ng in spatial learning and synaptic plasticity [19]. Hippocampal dysfunction has also been associated with rs12807809. In a previous study with 112 healthy volunteers, a relative reduction in hippocampal activation was observed in rs12807809 TT homozygotes compared with that in C-carriers during the acquisition phase of a contextual fear paradigm [20]. The hippocampus is a major component of the brain and plays important roles in learning and memory. Additionally, the hippocampus is the beginning and end of the Papez circuit [21] and an important component of the limbic system.

Hippocampal dysfunction in SZ has been reported in previous magnetic resonance imaging (MRI) studies. Structural studies of SZ have shown abnormalities in hippocampal volume and shape [22–24]. Structural abnormalities in the hippocampal formation have also been reported in populations at a high risk for psychosis [25, 26], implicating genetic susceptibility in hippocampal structural alterations in SZ. Additionally, resting-state functional MRI (fMRI) studies have reported increased hippocampal activation in individuals with SZ [27, 28].

To our knowledge, the association between SNP rs12807809 and hippocampal function in patients with SZ has not been studied. Functional connectivity (FC) is the temporal dependency between spatially remote neurophysiological events [29]. FC can describe the correlation between the time series of anatomically separate brain regions, reflecting the level of functional communication between different regions [29]. In the present study, we investigated the association between rs12807809 and resting-state hippocampal FC in adolescents and adults with SZ and healthy controls (HCs). We hypothesised that rs12807809 would be associated with abnormal hippocampal FC in SZ.

## Method

### Participants

We recruited 59 patients with SZ (mean age 21.70 ± SD 9.16 years, range 11–51 years, 73% female) from the outpatient clinic at the Department of Psychiatry, the First Hospital of China Medical University, and the Mental Health Center of Shenyang. SZ was diagnosed by 2 associate professors using the Diagnostic and Statistical Manual of Mental Disorders-IV (DSM-IV) criteria. Scores on the Brief Psychiatric Rating Scale (BPRS) were obtained from each participant except for one who did not complete these evaluations. In addition, 99 HC participants (mean age 23.87 ± SD 5.66 years, range 11–50 years, 58% female) without any history of mental illness or a family history of psychosis were recruited from the surrounding communities. Most participants were Han Chinese (*n* = 137, 87% of total participants; *n* = 53, 90% of patients with SZ; *n* = 84, 85% of HCs), and the ethnicity of the other participants was distributed as follows: Manchu (n = 13, 8% of total participants; *n* = 4, 7% of patients with SZ; *n* = 9, 9% of HCs), the Hui nationality (*n* = 3, 2% of total participants; n = 3, 3% of HCs), the Mongol nationality (*n* = 3, 2% of total participants; *n* = 1, 2% of patients with SZ; *n* = 2, 2% of HCs), the Korean nationality (*n* = 1, 1% of total participants; n = 1, 2% of patients with SZ) and the Zhuang nationality (*n* = 1, 1% of total participants; *n* = 1, 1% of HCs). The exclusion criteria for both groups were as follows: a history of major physical diseases, particularly those that may be related to changes in cerebral tissue, such as diabetes, hypertension, or metastatic tumours; unstable physical conditions, such as severe asthma; a history of nervous system diseases, including major head injury (loss of consciousness lasting more than five minutes), cerebrovascular disease, epilepsy, brain tumours and neurodegenerative diseases; mental retardation autism or extensive developmental disorder; claustrophobia; and strong magnetic objects in the body. All participants signed informed consent after having a sufficient understanding of the study as approved by the Institutional Review Board of China Medical University.

### Genotyping

Genomic DNA was extracted from the venous blood of each participant. The genotypes of rs12807809 were confirmed by the Sanger sequencing technique. Large studies [3, 8, 30] found a low frequency of the CC genotype (less than 10% in both patients with SZ and HCs). In our study, the frequency of the CC genotype was 1.7% in patients with SZ and 12.1% in HCs. Furthermore, compared to individuals with the CC or CT genotypes, the TT genotype may have a higher risk of SZ. Thus, we pooled participants carrying at least one C allele and compared them with T-allele homozygotes. The participants were further divided into two groups as follows: the C-carrier group (29 SZ, 46 HCs; mean age = 23.95 ± 6.73 years, 71% female) and the TT group homozygous for the risk T allele (30 SZ, 53 HCs; mean age = 22.25 ± 7.58 years, 57% female). The genotype frequencies were in accord with Hardy-Weinberg equilibrium (SZ: χ^2^ = 3.732, *P* = 0.053; HC: χ^2^ = 2.893, *P* = 0.089) (Additional file 1: Table S1). The minor allele frequencies were 25%.

### Image acquisition and processing

MRI data were collected by utilising a Signa 3.0 T MRI scanner. Head motion was reduced using foam pads. The participants were required to close their eyes and relax but remain awake. The scanning parameters of the resting-state fMRI were as follows: repetition time = 2000 ms, echo time = 30 ms, flip angle = 90°, field of view = 24 cm × 24 cm, matrix = 64 × 64. 35 axial slices were acquired with a slice thickness of 3 mm and no gap.

Resting-state fMRI data were preprocessed using the Data Processing Assistant for Resting-State fMRI (DPARSF 2.3, Advanced edition) toolbox [31]. After removing the first ten time points, the following steps were performed: slice timing correction, head motion correction (head motion parameters were calculated by evaluating displacement in each flat and the angular turn around each axis for each volume. The participants were excluded if head motion had a displacement of more than 3 mm in one or more flats or a 3° turn around one or more axes between each image. We excluded two patients with SZ and two HCs because of excessive head motion). Spatial normalisation was carried out by employing a standard EPI template from the Montreal Neurological Institute (MNI). The data were resampled to a voxel size of 3 × 3 × 3 mm^3^. A 6-mm full-width at half-maximum (FWHM) Gaussian filter was used for the spatial smoothing. Then, further preprocessing steps including linear detrending of physiological noise drift, low-frequency filtering (0.01–0.08 Hz) and nuisance covariates regression were performed. The nuisance covariates regression eliminated head motion parameters, white matter signal, cerebrospinal fluid signal and global mean signal.

### FC analysis

We defined the bilateral hippocampus as the region of interest (ROI) by using the automated anatomical labelling (AAL) atlas [32] included in REST (REST, V1.8), which was resampled to 3 × 3 × 3 mm^3^. For each participant, we computed the average time course for the hippocampal ROI at the voxel level. Voxel-wise correlation analyses between the hippocampal ROI and other cerebral regions were carried out to generate the FC map. The correlation coefficient map was converted into a z map by Fisher’s r-to-z transformation to improve normality.

### Statistical analysis

We used the chi-square test, independent sample t-test and analysis of variance to examine differences in sex, age, and illness duration among the 4 groups. All statistical analyses were performed using SPSS 22.0 software (SPSS Inc.). All statistical thresholds were set at *P* value < 0.05.

The main effect of diagnosis, genotype, and their interaction was assessed using a two-way analysis of covariance in Statistical Parametric Mapping 8 (SPM8) with diagnostic group (SZ and HC) and genotype group (CC/CT and TT) as the between-subject factors and sex and age as covariates. Statistical significance was defined as a corrected *P* < 0.05, with uncorrected thresholds of *P* < 0.001 and cluster size > 22 voxels as determined by Gaussian random field (GRF). The FC values of significant regions of interaction were extracted, and the Bonferroni correction was used for post hoc comparisons with a *P* < 0.05 considered the threshold for significance. Correlational analyses were performed to determine the correlation of FC values between regions showing significant differences with the BPRS scores in the two diagnostic groups.

## Result

### Participant characteristics

There was no significant difference in age (F_3,154_ = 0.137, *p* = 0.712) between the diagnostic and genotype groups. Similarly, there were no significant sex differences between SZ and HC (χ^2^ = 3.727, *p* = 0.054), between the genotype groups (χ^2^ = 3.343, *p* = 0.067), or between the genotype groups within each diagnostic group (χ^2^ = 2.054, *p* = 0.152 in the HC group and χ^2^ = 1.193, *p* = 0.275 in the SZ group). Within the SZ group, no significant difference was found in the duration of illness between the genotype groups (t = − 0.286, *p* = 0.776) (Table 1).

**Table 1:** Demographics and clinical data of participants

	SZ (*n* = 59)		HC (*n* = 99)			*P*
	CC/CT (*n* = 29)	TT (*n* = 30)	CC/CT (*n* = 46)	TT (*n* = 53)		
Age (years), mean ± SD	22.86 ± 8.99	20.57 ± 9.34	24.63 ± 4.80	23.21 ± 6.28	F = 0.137	0.712
Gender (male/female)	6/23	10/20	16/30	26/27	χ^2^ = 3.727	0.054 (SZ vs. HC)
					χ^2^ = 3.343	0.067 (CC/CT vs. TT)
					χ^2^ = 2.054	0.152 (HC:CC/CT vs. TT)
					χ^2^ = 1.193	0.275 (SZ:CC/CT vs. TT)
Duration of illness (months), mean ± SD ^a^	19.61 ± 27.94	22.38 ± 39.40	N/A	N/A	t = −0.286	0.776
Medication (yes/no)	25/4	25/5	N/A	N/A		
BPRS score, mean ± SD ^b^	32.69 ± 13.14	33.21 ± 9.66	18.42 ± 0.95	18.2 ± 0.64		

*SZ* Schizophrenia, *HC* Healthy controls, *SD* Standard Deviation, *BPRS* Brief Psychiatric Rating Scale

^a^information is missing for 8 patients. ^b^ information is missing for 1 patient

### FC analyses

The main effect of diagnosis, the main effect of genotype and the diagnosis by genotype interaction on FC are listed in Table 2.

**Table 2:** Clusters exhibiting the influence of groups and genotypes on FC

Brain area	Cluster size	Peak MNI coordinates			Peak F value
		X	Y	Z	
*Main effect of diagnostic groups*					
A. Left fusiform gyrus/left lingual gyrus/left inferior temporal gyrus	782	−42	−51	−12	30.30
B. Right lingual gyrus/right fusiform gyrus	76	21	−57	−3	17.81
C. Left caudate nucleus	38	−9	9	3	26.41
D. Left thalamus/right thalamus	53	6	−6	0	24.48
E. Left anterior cingulate gyrus/right anterior cingulate gyrus	70	0	21	21	16.64
*Diagnostic groups × genotype interaction*					
Left anterior cingulate gyrus/left middle cingulate gyrus/right middle cingulate gyrus	101	0	6	33	23.21

These findings correspond to a corrected *P < 0.05* by GRF correction. *BA* Brodmann’s area. Cluster size is in mm^3^

**Fig. 1:**
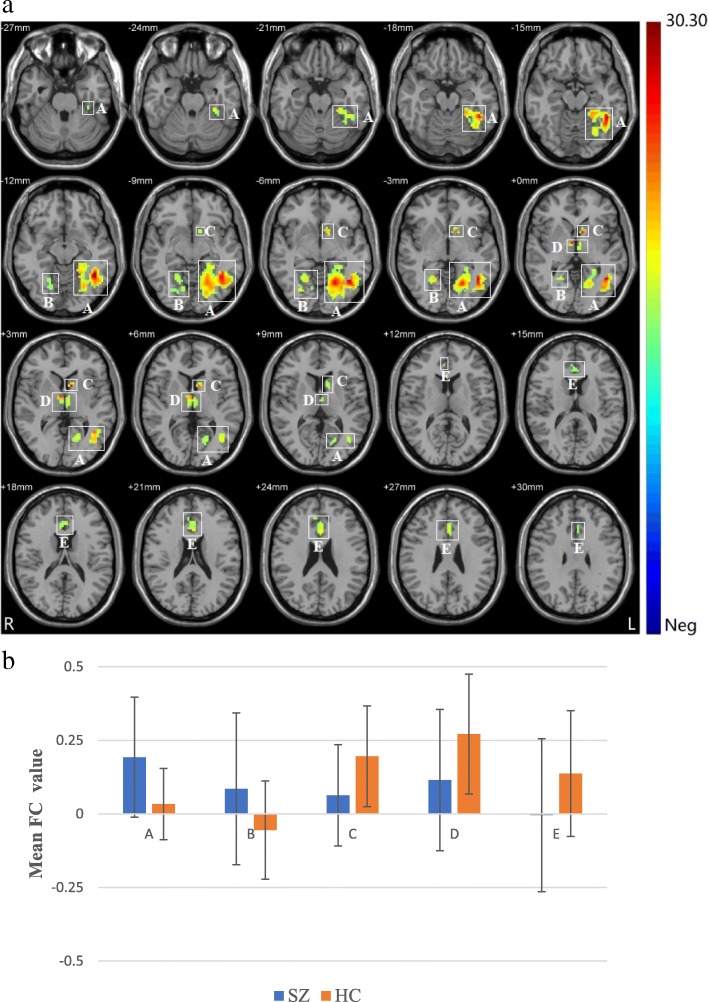
The main effect of diagnostic group. (**a**) Regions (white box) with main effect of diagnostic group include (**a**) left fusiform gyrus, left lingual gyrus, left inferior temporal gyrus, (**b**) right lingual gyrus, right fusiform gyrus, (**c**) left caudate nucleus, (**d**) left thalamus, right thalamus, (**e**) left anterior cingulate gyrus, right anterior cingulate gyrus (cluster-level threshold of *p* < 0.05 after GRF correction and cluster size =35). The coloured bar represents the range of F values. (**b**) Shown here are the FC values (mean ± standard deviation) extracted from regions with main effect of diagnostic group. The Y-axis represents FC values. The X-axis represents regions with main effect of diagnostic group

Significant main effects of diagnosis were discovered in FC between the hippocampus and bilateral fusiform gyrus, bilateral lingual gyrus, left inferior temporal gyrus, left caudate nucleus, bilateral thalamus and bilateral anterior cingulate gyri (Fig. 1). Compared to HCs, patients with SZ showed significantly increased FC between the hippocampus and bilateral fusiform gyrus, bilateral lingual gyrus, and left inferior temporal gyrus. However, compared to HCs, patients with SZ showed significantly decreased FC between the hippocampus and left caudate nucleus, bilateral thalamus and bilateral anterior cingulate gyri. There was no significant main effect of genotype.

**Fig. 2:**
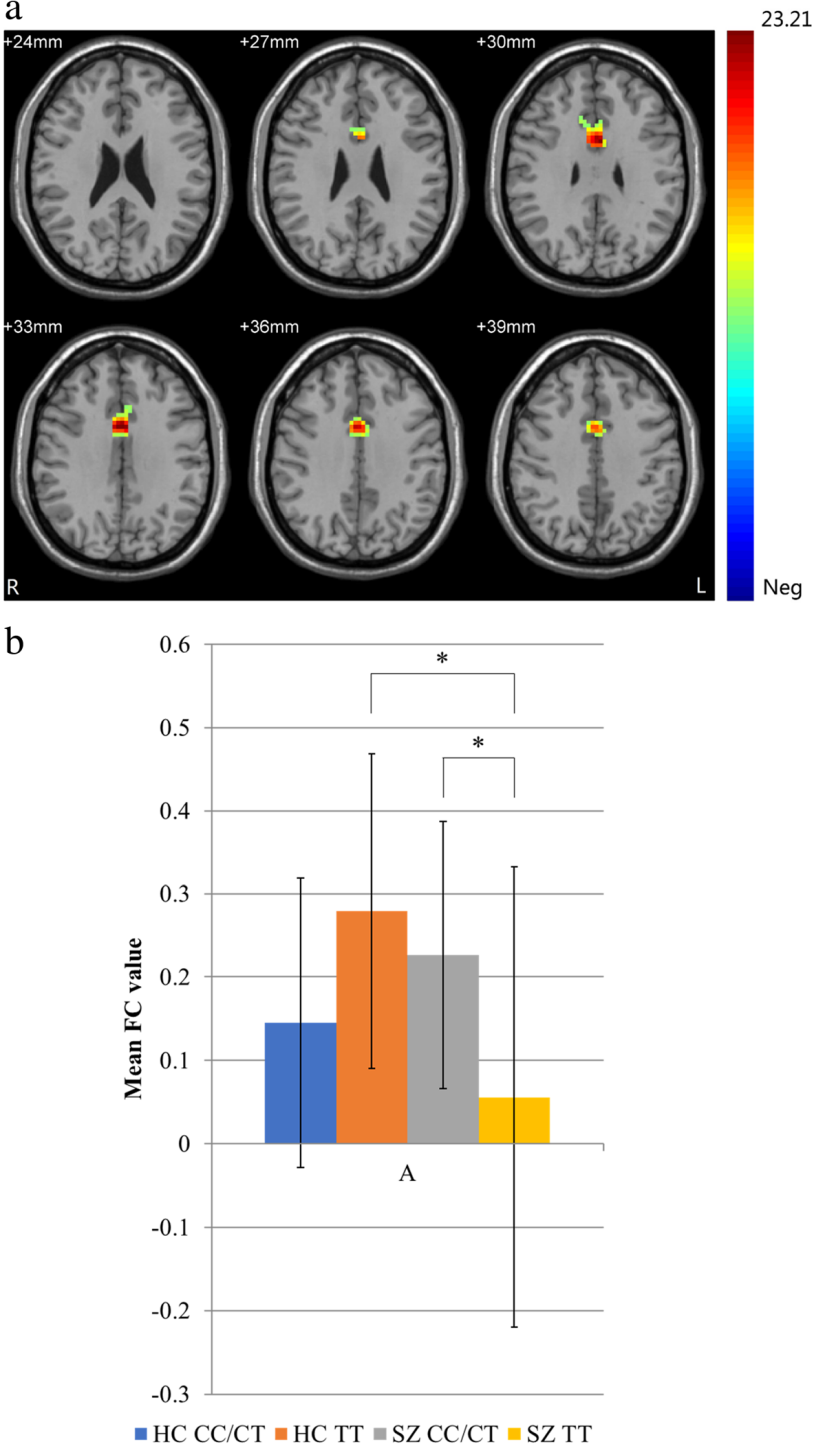
Diagnosis by genotype interaction. (**a**) Significant regions of diagnosis by genotype interaction from two-way ANCOVA include the bilateral middle cingulate gyri and left anterior cingulate gyrus (*P* < 0.05 by GRF correction and 22 voxels minimum). The coloured bar represents the range of F values. (b) Shown here are the mean (±standard deviation) FC values extracted from significant regions of the diagnosis by genotype interaction. Post hoc two-sample t-tests show that TT homozygotes with SZ have significantly lower FC values than do healthy TT homozygotes and C-carriers with SZ. *, *P* < 0.05

A significant diagnosis by genotype interaction was observed in FC between the hippocampus and bilateral middle cingulate gyri and left anterior cingulate gyrus (Fig. 2). Among patients with SZ, the TT group had significantly lower FC values than the C-carrier group. Within-genotype comparisons showed that TT homozygotes with SZ had a significantly lower FC than HCs who were TT homozygotes.

Correlation analyses between FC and BPRS scores showed no significant associations in the SZ group.

## Discussion

Our study yielded several significant results. First, significantly increased FC between the hippocampus and inferior temporal gyrus, lingual gyrus and fusiform gyrus was observed in patients with SZ compared to HCs. Second, FC was significantly decreased between the hippocampus and caudate nucleus, thalamus and anterior cingulate gyrus in the SZ group compared to the HC group. Third, the lowest FC between the hippocampus and anterior cingulate cortex was found in TT homozygotes with SZ compared to C-carriers with SZ and HC TT homozygotes. These results suggest that the rs12807809 TT genotype in the NRGN gene is associated with abnormal hippocampal FC at rest in SZ.

Compared to HCs, SZ patients exhibited increased FC between the hippocampus and fusiform gyrus, lingual gyrus, and inferior temporal gyrus. The increased FC between the hippocampus and lingual gyrus is consistent with previously reported changes in a longitudinal resting-state fMRI study [33]. Kraguljac et al. reported significantly increased left posterior hippocampal FC with the lingual gyrus in 34 drug-naïve patients with SZ compared to 34 HCs. The lingual gyrus is involved in visual processing and encoding visual memories [34]. The disconnect between the lingual gyrus and hippocampus in patients with SZ may be associated with an impairment of visual memory. Moreover, in the present study, increased FC was observed between the hippocampus and inferior temporal gyrus and fusiform gyrus. Few studies have reported abnormal FC between the hippocampus and inferior temporal gyrus and fusiform gyrus in patients with SZ. The fusiform gyrus participates in the within-category identification, word recognition, face and body recognition, and processing of colour information [34]. The main function of the inferior temporal gyrus is receiving information and object recognition [35]. The inferior temporal gyrus, fusiform gyrus and hippocampus work together to produce an understanding of the physical world. Abnormal FC between the hippocampus and fusiform gyrus and inferior temporal gyrus may be associated with disorientation in patients with SZ.

Decreased FC between the hippocampus and caudate nucleus, thalamus and anterior cingulate gyrus was found in the SZ group compared to that in the HC group. This decreased FC is consistent with some [36, 37] but not all [33] previous fMRI studies, indicating that hippocampal FC changes in patients with SZ without regard to genetic influences. Samudra et al. reported reduced hippocampal FC with the thalamus in 88 patients with psychosis compared to that in 65 HCs. Furthermore, anterior and posterior hippocampal ROI analyses showed reduced connectivity with the anterior cingulate cortex and thalamus. In another longitudinal study of 68 patients with first-episode SZ and 62 HCs, decreased FC with the cingulate cortex in the bilateral hippocampus network was found in patients compared with that in healthy controls at both baseline and follow-up. However, Kraguljac et al. reported significantly increased left anterior hippocampal FC with the caudate nucleus and anterior cingulate cortex in 34 drug-naïve patients with SZ compared to that in 34 HCs. The differences in the results among these studies may be associated the effect of antipsychotics. The Papez circuit was proposed by James Papez in 1937 [38] and involves various areas of the limbic system, such as the hippocampus and cingulate gyrus and the anterior thalamic nucleus [21]. The Papez circuit, which is believed to be involved in memory, specifically spatial and episodic memory [39, 40], is damaged in patients with SZ. Although our previous studies reported dysfunction of the Papez circuit in major depressive disorder [41], few studies have shown the function of the Papez circuit in SZ thus far. Our findings suggest that the Papez circuit may be involved in the pathophysiology of SZ. However, more effort is necessary to elucidate the mechanisms by which Papez circuit deficits are involved in the pathophysiology of SZ.

Although no difference was found in hippocampal FC between genotype groups, the lowest FC between the hippocampus and anterior cingulate gyrus was observed in TT homozygotes with SZ compared to that in C-carriers with SZ and HC TT homozygotes. In SZ studies, Ohi and colleagues also reported reduced grey matter volume in the left anterior cingulate cortex in TT homozygotes compared to that in C-carriers [5]. In addition, an fMRI study including 94 healthy participants showed stronger activation in the anterior cingulate cortex in TT homozygotes than in C-carriers during episodic memory encoding [42]. NRGN has the potential to influence hippocampal plasticity, spatial learning and long-term potentiation. Moreover, the anterior cingulate cortex participates in both cognitive and emotional processing [43, 44]; this structure is mainly involved in the modulation of attention or executive functions, error detection, working memory, episodic memory, the regulation of emotional responses, and in the salience of emotional and motivational information [43–47]. Both the anterior cingulate cortex and hippocampus are parts of the Papez circuit and are involved in episodic memory encoding. Therefore, our finding of abnormal FC between the hippocampus and anterior cingulate cortex suggests that the T risk allele may influence the Papez circuit deficits observed in SZ.

The current study has some limitations. First, the sample size was not large enough; thus, a false positive result may have been obtained. Therefore, a study with a larger sample should be conducted to further assess the effect of genes. Second, other SNPs in this gene or other genes involved in SZ were not included in our study. Third, we did not consider the effect of antipsychotics and the course of the disease in patients with SZ. Further studies should be conducted in first-episode, drug-naïve patients with SZ.

## Conclusion

In conclusion, for the first time, the present study verifies that the NRGN rs12807809 TT genotype may be associated with Papez circuit dysfunction in patients with SZ. This finding may implicate NRGN variations and Papez circuit dysfunction in SZ pathophysiology.

## Additional file

Additional file 1: Supplementary materials. (DOCX 14 kb)

## Abbreviations

BPRS: Brief Psychiatric Rating Scale
CaM: Calmodulin
FC: Functional connectivity
HC: Healthy control
Ng: Neurogranin
PKC: Protein kinase C
ROI: Region of interest
SZ: Schizophrenia
