# Supplementary material for: Association between NRGN gene polymorphism and resting-state hippocampal functional connectivity in schizophrenia
Source: BMC Psychiatry. 2019 Apr 5;19:108. doi: 10.1186/s12888-019-2088-5 (PMC6451258; doi:10.1186/s12888-019-2088-5)
Supplement: Supplementary file 1 — Supplementary materials. (DOCX 14 kb) [file 12888_2019_2088_MOESM1_ESM.docx]

| Table S1. Allele and genotype frequencies of NRGN rs12807809 | | | | | | | |
| --- | --- | --- | --- | --- | --- | --- | --- |
|  | Genotype Distribution (%) | | | Allelic Distribution (%) | | χ^2^ | HWE *p* Value |
|  | CC | CT | TT | C | T |  |  |
| SZ(n=59) | 1.7 | 47.5 | 50.8 | 25.4 | 74.6 | 3.732 | 0.053 |
| HC(n=99) | 12.1 | 34.3 | 53.5 | 29.3 | 70.7 | 2.893 | 0.089 |
| SZ, schizophrenia; HC, healthy controls. | | | | | | | |

| Table S2. Effect of diagnostic groups, genotype and diagnostic group × genotype on mean FC value of region of significant ANCOVA results. | | | | | | |
| --- | --- | --- | --- | --- | --- | --- |
| Region of significant ANCOVA results | diagnostic groups | | genotype | | diagnostic groups × genotype | |
|  | β | 95%CI | β | 95%CI | β | 95%CI |
| *Main effect of diagnostic groups* |  |  |  |  |  |  |
| A. Left fusiform gyrus/left lingual gyrus/left inferior temporal gyrus | 0.107 | 0.033-0.180 | -0.031 | -0.093 to 0.031 | 0.101 | 0.000-0.203 |
| B. Right lingual gyrus/right fusiform gyrus | 0.114 | 0.018-0.210 | 0.013 | -0.069 to 0.095 | 0.052 | -0.081 to 0.186 |
| C. Left caudate nucleus | -0.16 | -0.240 to -0.081 | 0.027 | -0.041 to 0.095 | 0.057 | -0.053 to 0.168 |
| D. Left thalamus/right thalamus | -0.166 | -0.269 to -0.064 | 0.004 | -0.084 to 0.091 | 0.019 | -0.123 to 0.161 |
| E. Left anterior cingulate gyrus/right anterior cingulate gyrus | -0.043 | -0.15 to 0.064 | 0.095 | 0.004-0.186 | -0.189 | -0.338 to -0.04 |
| *Diagnostic groups × genotype interaction* |  |  |  |  |  |  |
| Left anterior cingulate gyrus/left middle cingulate gyrus/right middle cingulate gyrus | 0.082 | -0.012 to 0.175 | 0.134 | 0.055-0.214 | -0.305 | -0.435 to -0.175 |
